# Effectiveness of Restylane Lyft for Nonsurgical Full Nose Correction in Chinese Patients: A Retrospective 3-Dimensional Imaging-Based Cohort Study

**DOI:** 10.1093/asjof/ojag037

**Published:** 2026-02-24

**Authors:** Zhiqiang Xue, Changwon Kim, Dahee Hwang, Haiyan Cui

## Abstract

**Background:**

In Chinese patients, common deficiencies—shortened columella, underprojected tip, and wide alar base—reflect maxillonasal dysplasia. Although Restylane Lyft is effective for dorsum augmentation, its role in comprehensive reshaping is less defined.

**Objectives:**

The aim of this study was to assess the efficacy and safety of Restylane Lyft for full nasal contour correction using a modified injection technique targeting the nasal spine and septal structures, evaluated by 3-dimensional imaging.

**Methods:**

Thirty-six patients (32 women, 4 men; mean age 31 years) underwent augmentation with Restylane Lyft. Injections were placed supraperiosteally at the radix, dorsum, and canine fossa, with a deep bolus at the columellar base above the nasal spine and precise deposition between the anterior septal angle and interdomal ligament to elongate the sub-tip lobule. Volumetric and morphologic outcomes were assessed at baseline, 1, 3, 6, and 12 months. Primary endpoints included nasal volume, tip projection, columellar height, and alar width. Satisfaction and adverse events were documented.

**Results:**

Posttreatment, nasal volume increased +1.2 cm^3^ and tip projection +2.2 mm, with elongation of +2.5 mm and no widening. At 12 months, improvements persisted (+0.7 cm^3^, +1.2 mm). Satisfaction was high, with only mild, self-limiting adverse events and no ischemic complications.

**Conclusions:**

Restylane Lyft, applied through precise anatomical targeting, may offer effective and safe correction of full nasal contours in Chinese patients. Midline bolus at the nasal spine and septal space injections seems to allow for tip projection and lobular elongation without compromising tip definition. More extensive studies need to be conducted to confirm these finding.

**Level of Evidence: 4 (Therapeutic):**

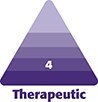

Nasal morphology plays a central role in facial balance, particularly among East Asian populations, where characteristic features include a low nasal dorsum, flat radix, wide alar base, and short columella. These traits are often linked to relatively deficient osteocartilaginous support at the anterior border of the piriform aperture and are considered manifestations of maxillonasal hypoplasia or dysplasia in certain subsets of patients.^1,2^ Although surgical rhinoplasty using autologous cartilage grafts remains the standard of care for structural nasal correction, it involves significant procedural invasiveness, donor-site morbidity, longer recovery times, and higher cost. These limitations have increasingly motivated patients to seek less invasive, office-based alternatives.^3,4^

Hyaluronic acid (HA) filler–based nonsurgical rhinoplasty has gained considerable popularity over the past decade because of its safety profile, reversibility, and minimal downtime. When performed with appropriate anatomical knowledge and technique, it offers reliable outcomes, particularly in augmentation rhinoplasty cases. Expert consensus emphasizes several key principles to ensure safe and effective outcomes: the use of fillers with high-elastic modulus and cohesivity, deep periosteal or perichondrial injection planes, and low-volume, slow-deposit bolus technique to minimize vascular compromise.^5,6^ These recommendations are especially pertinent in nasal regions where the vascular network is complex and the margin for error is narrow.^7,8^

Despite these advancements, most existing studies on nonsurgical rhinoplasty have focused on dorsal augmentation alone, with relatively little quantitative analysis of volumetric or angular outcomes in the nasal tip and columella. Moreover, evaluations in the current literature are predominantly based on 2-dimensional photography or subjective aesthetic scales, lacking objective morphometric data.^9,10^ In contrast, the development of high-resolution 3-dimensional (3D) imaging technologies now permits precise, reproducible quantification of changes in nasal projection, volume, and angular relationships. Commercially available systems such as Vectra, Artec, and LifeViz have demonstrated sub-millimeter accuracy and are increasingly integrated into both preoperative planning and postoperative outcome assessment.^11,12^

Although several studies have examined nasofrontal or nasolabial angle changes following HA rhinoplasty, few have concurrently documented full-unit nasal modifications encompassing the dorsum, tip, and columella. For example, Chen et al reported accurate simulation of aesthetic outcomes using 3D analysis, but their study primarily addressed nasofrontal and nasolabial angular change without longitudinal analysis of tip or columellar projection.^13^

Given these gaps in the literature, the present study aims to investigate whether a structured, anatomically guided HA injection protocol—targeting the radix, nasal dorsum, anterior septal angle, and columellar base—can achieve objective, durable, and reproducible 3D nasal enhancements in a Chinese population. We hypothesize that deep-layer injections using Restylane Lyft, a filler with favorable rheological properties for structural contouring, can produce quantifiable improvements in nasal projection and volume without undesirable lateral spreading or broadening of the nasal tip.^14,15^ By employing a validated 3D scanning protocol, we aim to provide high-fidelity morphometric data over a 12-month follow-up period, thereby contributing evidence-based insights into full-unit, nonsurgical nasal reshaping in East Asian patients.

## METHODS

### Study Design and Patient Selection

This retrospective observational study was conducted at a single private aesthetic clinic in Beijing, China, and was approved by the institutional ethics committee (approval number: SECCR2024-08-01). Patients who underwent nonsurgical rhinoplasty using HA fillers between January 2022 and January 2023 were eligible for inclusion. Criteria included Chinese ethnicity, age between 20 and 50 years, and structural nasal deficiencies such as underprojected tip, short columella, or low dorsum. Patients with previous nasal surgery or dermal filler treatment within the preceding 12 months were excluded.

Additional exclusion criteria comprised pregnancy, lactation, known hypersensitivity to HA or lidocaine, coagulation disorders, autoimmune disease, active facial skin infection, or history of vascular complications from dermal fillers. All patients provided written informed consent for both treatment and the use of anonymized clinical data and imagery.

### Injection Technique

All procedures were performed by a single board-certified plastic surgeon with over 10 years of experience in facial filler techniques. After antiseptic cleansing with 0.5% chlorhexidine, injections were performed using Restylane Lyft (Q-Med, Uppsala, Sweden), a NASHA-based HA filler with high-elastic modulus and lidocaine.

**Figure 1.**
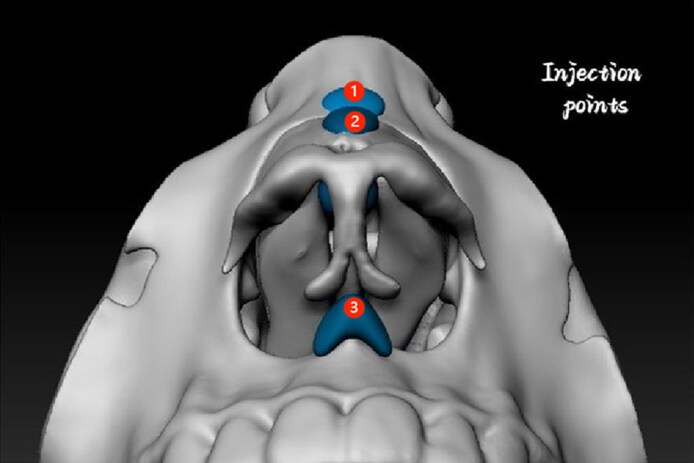
Head-up position illustration of the injection points: (1) radix, (2) nasal dorsum, (3) columella base.

**Figure 2.**
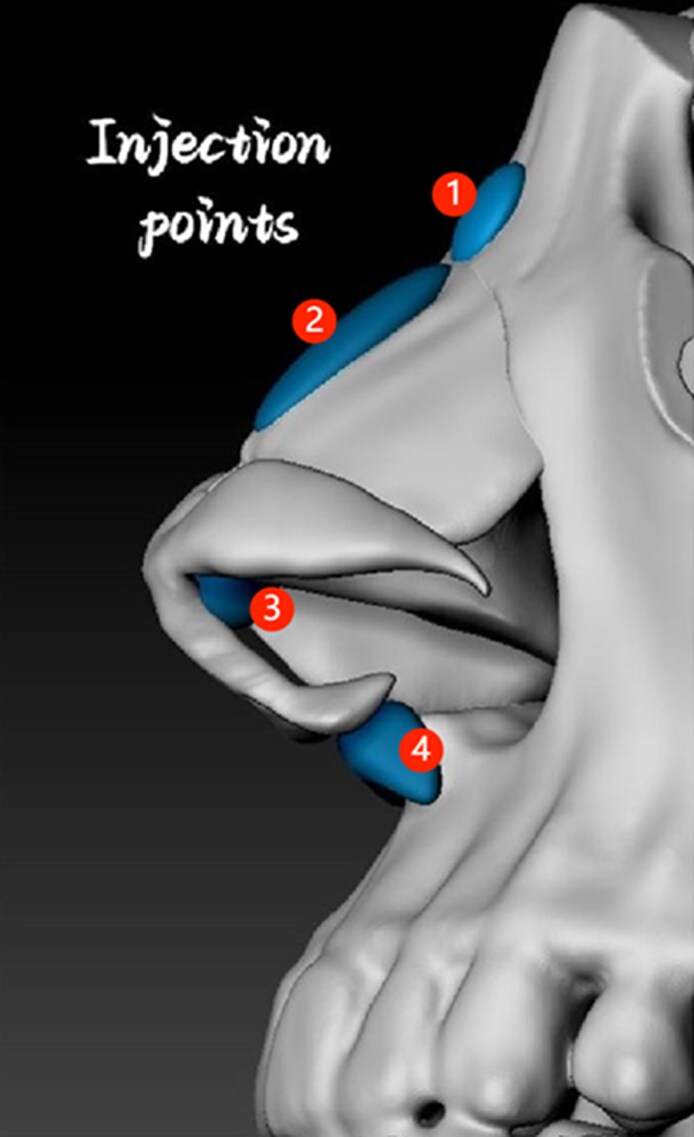
Lateral illustration of the injection points: (1) radix, (2) nasal dorsum, (3) nasal tip, (4) columella base.

A 1 mL prefilled syringe attached to a 27G half-inch needle was used for direct injection. Injections were performed strictly in the midline and followed a standardized sequence: (1) Radix (nasofrontal angle): 0.1 to 0.2 mL supraperiosteal bolus; (2) nasal dorsum: 0.2 to 0.4 mL linear threading or serial boluses along the cartilaginous dorsum; (3) anterior septal angle (tip support): 0.3 to 0.4 mL deep depot bolus; and (4) columellar base (nasal spine): 0.4–0.5 mL supraperiosteal bolus. Injection points are illustrated in Figures 1 and 2. Aspirations were performed before each injection. Gentle molding was applied postinjection to maintain alignment and prevent lateral dispersion. Injection points, tissue planes, and mean filler volumes used for nonsurgical rhinoplasty are listed in Table 1. More specifically, we have provided the detailed injection process in the Video.

**Table 1.** Injection Points, Tissue Planes, and Mean Filler Volumes Used for Nonsurgical Rhinoplasty

	Radix	Dorsum	Columella base	Tip
Layer	Above periosteum	Above periosteum	Above periosteum	The space between interior angle of septum and interdomain ligament
Dose (mean ± SD), mL	0.15 ± 0.03	0.35 ± 0.07	0.46 ± 0.09	0.38 ± 0.08

Summary of standardized injection sites, anatomical layers, and mean filler volumes administered per region. All injections were performed in the midline using a 30G needle with aspiration before each bolus. Volumes represent the mean ± standard deviation (SD) of Restylane Lyft (Galderma Laboratories) injected at each anatomical landmark.

### Three-Dimensional Imaging and Morphometric Analysis

**Figure 3.**
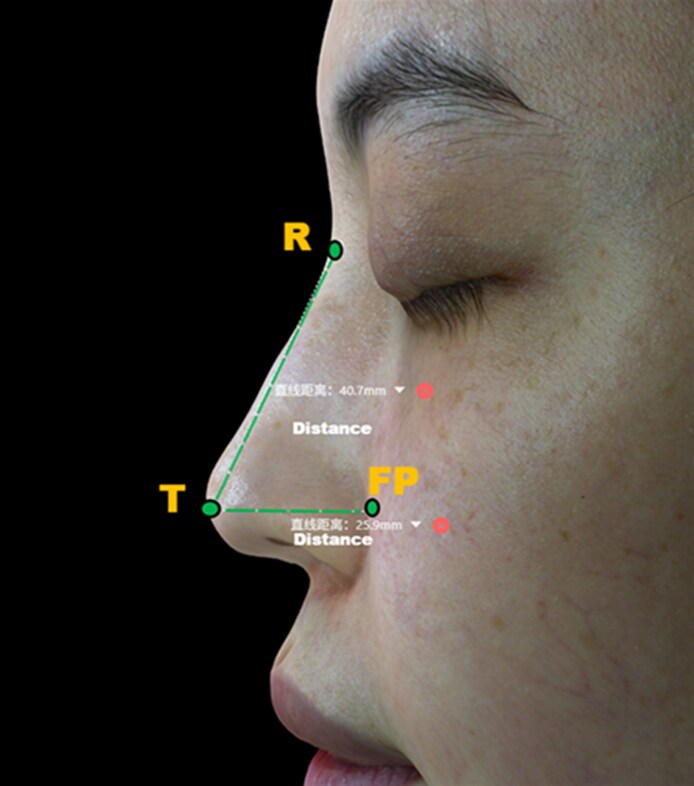
Illustration of the measurement of tip projection and tip elongation in Mirage3DAI imaging system (Hangzhou Xiaofu Technology Co., Ltd). “R” refers to the radix point; “T” refers to nasal tip point; “FP” refers to facial plane.

**Figure 4.**
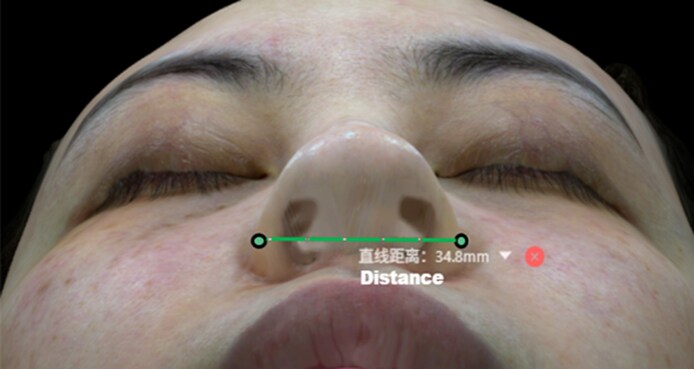
Illustration of the measurement of alar width (the distance between the most lateral points of the alar) in Mirage3DAI imaging system (Hangzhou Xiaofu Technology Co., Ltd).

Three-dimensional imaging was performed using the Meiji P2 3D scanner (Hangzhou Xiaofu Technology Co., Ltd, Hangzhou, China), with sub-millimeter accuracy (≤0.1 mm) under controlled lighting and neutral facial expression. Scans were acquired at baseline, right after injection, and at 1, 6, and 12 months postprocedure. All images were processed and analyzed using the manufacturer's proprietary software. Quantitative morphometric measurements included: (1) Nasal volume (mm^3^): from nasion (n) to subnasale (sn); (2) Tip projection (mm): defined as the horizontal distance from the alar crease of the facial plane to the tip-defining point (TP) on lateral view, measuring the extent to which the tip protrudes along the sagittal axis relative to a vertical reference plane; (3) Tip elongation (mm): straight-line distance from the nasion (N) to the pronasale (Prn) on 3D facial imaging.^16^ This parameter has been validated in recent 3D nasal antTip elongation studies, demonstrating excellent intra- and interrater reliability.^17^ Measurements of the tip projection and tip elongation in 3D simulated images are illustrated in Figure 3; (4) Alar width: defined precisely as the distance between bilateral alar base points on the coronal plane in the 3D dataset, confirming reproducible measurement accuracy.^18^ Measurement of alar width in 3D simulated images is illustrated in Figure 4. Each parameter was assessed independently by 2 trained raters blinded to the time point. Interobserver variability was assessed; discrepancies >1% were resolved by consensus review.

### Statistical Analysis

All statistical analyses were performed using SPSS version 27.0 (IBM Corp., Armonk, NY). Descriptive statistics were reported as means ± standard deviation. Repeated-measures analysis of variance with Bonferroni correction was employed to compare morphometric variables across follow-up intervals. A *P*-value of <.05 was considered statistically significant.

## RESULTS

### Three-Dimensional Morphometric Outcomes

A total of 38 patients (32 women and 6 men; mean age, 29.3 ± 5.4 years) completed all follow-up assessments through 12 months. All patients were Chinese adults presenting with midline nasal volume deficiencies consistent with mild-to-moderate maxillofacial hypoplasia.

**Figure 5.**
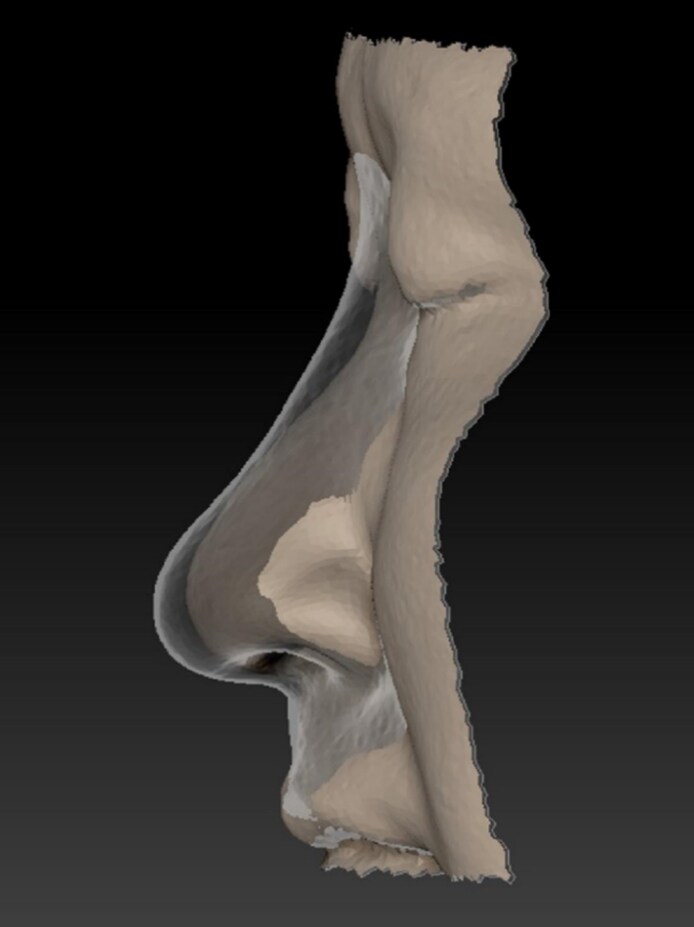
Lateral position illustration of shape changes right after injection (the transparent part) compared with the baseline (the brown part). Note the elevation of the nasal tip, elongation of the columella, and the change of nasolabial angle.

**Figure 6.**
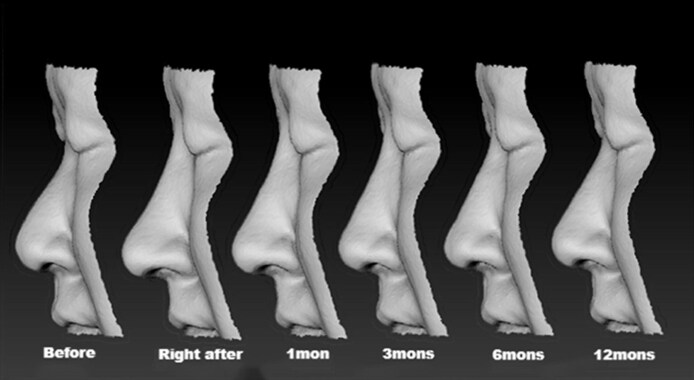
Sequential recording of the 3-dimentional data at every critical timing (before treatment, right after treatment, 1 month after, 3 months after, 6 months after, and 12 months after treatment). Note the decline of height in the area of radix is more obvious than in the nasal tip.

Objective analysis showed statistically significant improvement in nasal tip projection and total nasal volume following hyaluronic acid injection. Lateral position illustration of shape changes right after injection compared with the baseline is illustrated in Figure 5. Nasal tip projection increased from a baseline mean of 19.8 ± 2.3 to 23.7 ± 2.1 mm at 1-month posttreatment (*P* < .001). At 6 months, the mean projection was 23.2 ± 2.0 mm, and at 12 months, 22.4 ± 2.2 mm—both remaining significantly elevated compared with baseline (*P* < .001). Although a gradual decline was observed over time, projection did not return to pretreatment values. Total nasal volume, calculated from nasion to subnasale using 3D scan data, increased from 3.56 ± 0.45 cm^3^ at baseline to 4.32 ± 0.41 cm^3^ at 1 month (*P* < .001). This volume declined modestly to 4.11 ± 0.44 cm^3^ at 6 months and 3.95 ± 0.42 cm^3^ at 12 months, with all time points showing statistically significant differences from baseline (*P* < .001). Interrater reliability for volumetric and projection measurements was high, with intraclass correlation coefficients exceeding 0.95. Sequential recording of the 3D data at every critical timing is illustrated in Figure 6. Quantitative morphometric changes in nasal tip projection and volume following hyaluronic acid injection are summarized in Tables 2 and 3.

**Table 2.** Quantitative Morphometric Changes in Nasal Tip Projection and Volume Following Hyaluronic Acid Injection (*n* = 36)

Parameter	Baseline	1 Month	6 Months	12 Months	*P*-value vs baseline
Nasal tip projection (mm)	19.8 ± 2.31	23.7 ± 2.12	23.2 ± 2.01	22.4 ± 2.23	<.001
Total nasal volume (cm^3^)	3.56 ± 0.45	4.32 ± 0.41	4.11 ± 0.44	3.95 ± 0.42	<.001

Data are expressed as the mean ± standard deviation (SD). Statistical analysis performed using repeated-measures analysis of variance with Bonferroni correction.

All posttreatment values remained significantly higher than baseline (*P* < .001).

**Table 3.** Nasal Morphological Outcomes Following Hyaluronic Acid Injection Over 12 Months

Time point	Nasal volume change (cm^3^, mean ± SD)	Tip projection (mm, mean ± SD)	Alar width (mm, mean ± SD)	Tip elongation (mm, mean ± SD)
Baseline (right after injection)	+1.2 ± 0.1	+2.2 ± 0.2	−2.5 ± 0.2	+2.5 ± 0.2
1 month	+1.1 ± 0.1	+1.9 ± 0.1	−2.4 ± 0.2	+2.5 ± 0.2
3 months	+1.0 ± 0.1	+1.8 ± 0.1	−2.4 ± 0.2	+2.4 ± 0.2
6 months	+0.8 ± 0.1	+1.6 ± 0.1	−2.2 ± 0.2	+2.2 ± 0.2
12 months	+0.7 ± 0.1	+1.2 ± 0.1	−1.7 ± 0.1	+1.9 ± 0.2

Changes in nasal morphometric parameters measured by 3-dimensional imaging at defined postoperative intervals. Data represent mean change from baseline (preinjection) values. Nasal volume and tip projection demonstrated sustained enhancement over 12 months, whereas alar width showed a mild, consistent decrease. Tip elongation remained stable with gradual regression after 6 months.

### Patient Satisfaction Outcomes

Patient satisfaction was assessed using a 5-point Likert scale ranging from “very dissatisfied” to “very satisfied.” At 1-month posttreatment, 92.1% of patients (35/38) reported being “satisfied” or “very satisfied.” At 6 months, satisfaction remained high at 89.5% (34/38), and at 12 months, 81.6% (31/38) of patients maintained a positive satisfaction rating. None of the patients reported being “dissatisfied” or “very dissatisfied” at any time point. Three patients (7.9%) expressed a desire for minor retreatment after 12 months but declined additional filler at that time. Patient and investigator satisfaction scores are summarized in Table 4.

**Table 4.** Patient and Investigator Satisfaction Scores Following Nonsurgical Rhinoplasty

Assessment time point	Patient satisfaction (mean ± SD)	Investigator satisfaction (mean ± SD)
1 month	98 ± 3	98 ± 3
3 months	96 ± 2	95 ± 2
6 months	92 ± 2	95 ± 3
12 months	88 ± 2	90 ± 2

Mean satisfaction scores (0-100 scale) reported by patients and investigators at designated posttreatment intervals. Both groups demonstrated high satisfaction throughout the 12-month follow-up, with gradual decline corresponding to expected filler resorption. Investigator assessments remained consistently aligned with patient-reported outcomes.

Mean, average satisfaction score on a 0-100 visual analog scale.

### Durability and Safety

**Figure 7.**
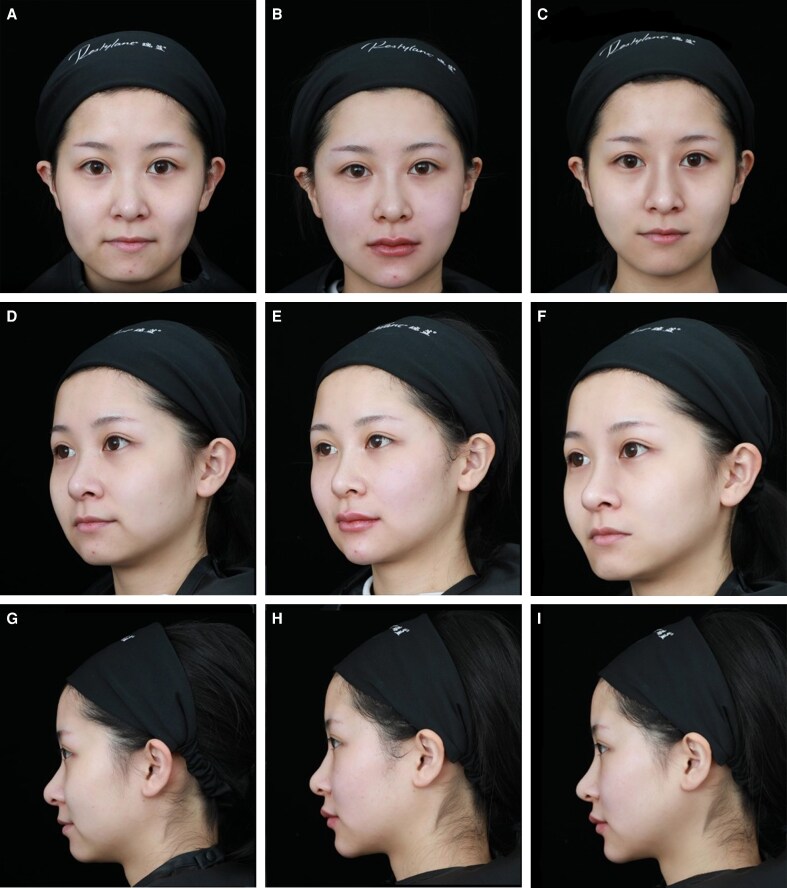
Comparison (A, D, G) before, (B, E, H) immediately after, and (C, F, I) 12 months after hyaluronic acid filler rhinoplasty. A 29-year-old female patient demonstrated noticeable enhancement of nasal dorsum definition and tip projection immediately following midline hyaluronic acid injection. Treatment was performed using Restylane Lyft (Galderma Laboratories, Lausanne, Switzerland) in a multilayered approach with supraperiosteal and interdomal placement. No complications were observed.

**Figure 8.**
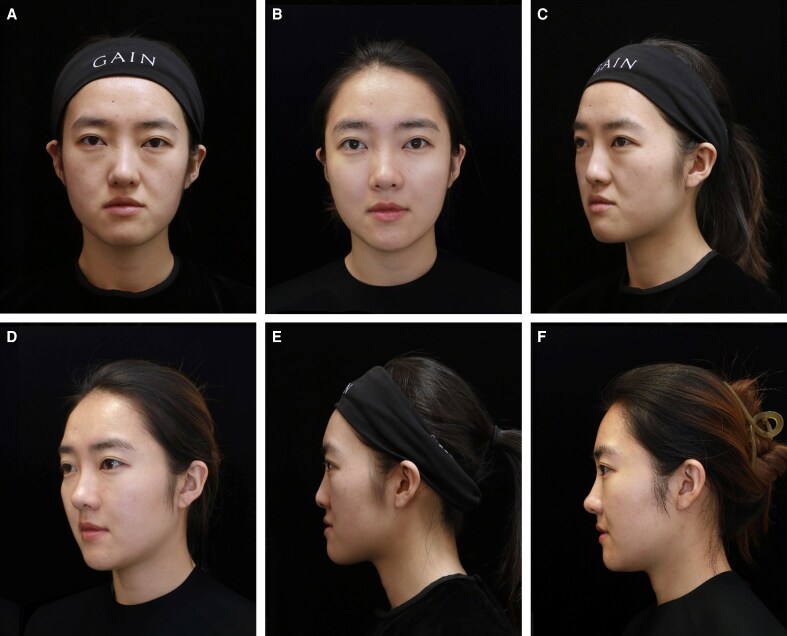
Comparison (A, C, E) before and (B, D, F) 12 months after hyaluronic acid filler rhinoplasty. A 32-year-old female patient demonstrated noticeable enhancement of nasal dorsum definition and tip projection immediately following midline hyaluronic acid injection. The usage amounts for each part are as follows: radix (0.2 mL); nasal dorsum (0.3 mL); nasal tip (0.4 mL); columella base (0.4 mL); canine fossa (0.6 mL).

Both projection and volume exhibited the greatest enhancement at 1 month, with gradual regression over time. Importantly, significant improvements were sustained through 12 months without the need for retreatment in the cohort studied (Figures 7, 8). No serious complications were reported. Mild transient edema or erythema occurred in 8 patients (21%) within 72 h postinjection, resolving spontaneously. No vascular adverse events, nodules, or prolonged inflammatory responses were observed. Treatment-related adverse events are summarized in Table 5.

**Table 5.** Treatment-Related Adverse Events Following Hyaluronic Acid Injection

Event type	No. of patients	Resolution time (mean days)
Swelling	6	3
Bruising	3	14
Nodule formation	2	14
Infection	0	0

Summary of treatment-related local adverse events observed during the 12-month follow-up period. All events were mild and transient, resolving spontaneously without medical intervention. No cases of vascular occlusion, necrosis, or infection were recorded. Mean days = average time to complete clinical resolution.

## DISCUSSION

This study confirms that Restylane Lyft provides durable volumetric enhancement and structural definition of the nasal tip and columella—anatomical regions often underserved by traditional nonsurgical techniques. Its high-elastic modulus (*G*′) and cohesivity contribute to sustained projection and contour integrity, consistent with previous rheologic analyses of soft-tissue fillers.^5,6^

All patients in this cohort were treated using a modified injection approach: a single deep bolus placed over the nasal spine, at the base of the columella, rather than the more widely adopted technique of linear injection along the columellar shaft. Mechanically, deep filler placement at the anterior septal angle and nasal spine provides a hinge-like effect, elevating the medial crura to enhance projection, akin to the structural support achieved with columellar strut grafts in rhinoplasty.^3^ Another consideration is that the midline supraperiosteal plane above the anterior nasal spine is known to be relatively avascular, in contrast to the columella shaft, which houses branches of the superior labial artery.^7,19,20^ In this study, the average increase in tip projection was 2.2 mm at baseline and remained 1.2 mm at 12 months, with no observed complications such as tip broadening or asymmetry. This approach demonstrated excellent structural support for nasal tip projection, with a significantly reduced risk of vascular complications. No ischemic events or vascular complications occurred in this series.

In addition to midline columellar base augmentation, a refined anatomical target was employed in the space between the anterior septal angle and the interdomal ligament. Filler placement at this junction yielded consistent elongation of the sub-tip lobule without widening the tip-defining points—a key aesthetic goal in nasal refinement. Jung demonstrated that dual-plane injection techniques targeting deep and superficial compartments allow precise nasal lengthening without tip bulbosity.^12^ Yu et al also reported reliable sub-tip elongation and elevation using this septal space approach, with improved columellar support and narrow tip definition.^21^

An additional strength of this study was the use of 3D facial scanning, which enabled objective and reproducible measurement of volumetric and spatial changes. In this study evaluating comprehensive nasal contour correction with Restylane Lyft, the Mirage3DAI 3D imaging system (by Hangzhou Xiaofu Technology Co., Ltd) provided significant advantages over conventional 3D scanners.^22^ Unlike general-purpose scanners primarily optimized for object modeling or broad facial morphometry, Mirage3DAI offers high-resolution, dermatology-grade facial imaging tailored for midline and micro-volumetric assessments. Its AI-assisted tracking algorithms and calibrated optical fidelity enabled precise quantification of subtle nasal changes, such as columellar elongation, tip projection, and soft-tissue migration postinjection—parameters often challenging to capture with standard photogrammetry or LiDAR-based tools.^23^

The system's multi-angle synchronization and automated alignment significantly reduced operator variability during follow-up assessments across multiple time points (1, 3, 6, and 12 months), ensuring consistency in measurement of volume (+1.2 cm³) and projection changes (+2.5 mm). Moreover, its clinical integration and intuitive workflow allowed for seamless patient data acquisition, rapid 3D reconstruction, and high patient compliance in a noninvasive manner. Compared with other 3D imaging modalities, Mirage3DAI enabled more granular and reproducible analysis of soft-tissue dynamics, directly supporting the study's objective assessment of nasal reshaping efficacy and long-term filler stability.

### Limitations

This study has several limitations. As a retrospective, single-center analysis without a control group, its findings may be limited by selection and observer biases. Future studies should incorporate prospective designs comparing midline nasal spine and septal angle injections vs traditional columellar or tip techniques. The generalizability of our findings should also be validated in more diverse patient populations. Long-term outcomes beyond 12 months, as well as dynamic assessments under facial expression, would offer greater insight into the structural longevity of the filler material. Additionally, future work could include Doppler-assisted mapping or imaging to further establish vascular safety in this anatomical corridor.

## CONCLUSIONS

Restylane Lyft, applied through precise anatomical targeting, may offer effective and safe correction of full nasal contours in Chinese patients. Midline bolus at the nasal spine and septal space injections seems to allow for tip projection and lobular elongation without compromising tip definition. More extensive studies need to be conducted to confirm these findings.
